# Assessment of Associations between Malaria Parasites and Avian Hosts—A Combination of Classic System and Modern Molecular Approach

**DOI:** 10.3390/biology10070636

**Published:** 2021-07-09

**Authors:** Xi Huang

**Affiliations:** MOE Key Laboratory for Biodiversity Science and Ecological Engineering, College of Life Sciences, Beijing Normal University, Beijing 100875, China; xi.huang@bnu.edu.cn

**Keywords:** avian haemosporidians, host-parasite association, infection intensity, molecular, quantitative PCR

## Abstract

**Simple Summary:**

Throughout history, frequent outbreaks of diseases in humans have occurred following transmission from animals. While some diseases can jump between birds and mammals, others are stuck to closely related species. Understanding the mechanisms of host–parasite associations will enable us to predict the outbreaks of diseases and will therefore be important to society and ecological health. For decades, scientists have attempted to reveal how host–parasite associations are formed and persist. The key is to assess the ability of the parasite to infect and reproduce within the host without killing the host. Related studies have faced numerous challenges, but technical advances are providing solutions and are gradually broadening our understanding. In this review, I use bird malaria and related blood parasites as a model system and summarize the important advances in techniques and perspectives and how they provide new approaches for understanding the evolution of host–parasite associations to further predict disease outbreaks.

**Abstract:**

Avian malaria and related haemosporidian parasites are responsible for fitness loss and mortality in susceptible bird species. This group of globally distributed parasites has long been used as a classical system for investigating host–parasite associations. The association between a parasite and its hosts can be assessed by the prevalence in the host population and infection intensity in a host individual, which, respectively, reflect the ability of the parasite to infect the host and reproduce within the host. However, the latter has long been poorly investigated due to numerous challenges, such as lack of general molecular markers and limited sensitivity of traditional methods, especially when analysing naturally infected birds. The recent development of genetic databases, together with novel molecular methodologies, has shed light on this long-standing problem. Real-time quantitative PCR has enabled more accurate quantification of avian haemosporidian parasites, and digital droplet PCR further improved experimental sensitivity and repeatability of quantification. In recent decades, parallel studies have been carried out all over the world, providing great opportunities for exploring the adaptation of haemosporidian parasites to different hosts and the variations across time and space, and further investigating the coevolutionary history between parasites and their hosts. I hereby review the most important milestones in diagnosis techniques of avian haemosporidian parasites and illustrate how they provide new insights for understanding host–parasite associations.

## 1. Introduction

The past decades have seen a growing interest in protozoan parasites due to their role in biological diversity and ecosystem health, and parallel studies have been carried out to investigate the associations between parasites and their hosts. During the transmission, the parasite may occasionally encounter novel hosts and establish novel associations if they are compatible with each other [1]. This process is known as host shift and is the main cause of emerging infectious diseases (EIDs) in humans [2,3], livestock [4], and wildlife [5].

In addition to the diversity of parasites, the compatibility between parasites and their hosts is a key element of disease risk [6]. A host–parasite association can be formed only when the parasite can complete its life cycle within the host and be transmitted, which requires a minimum parasite intensity, below which the parasite cannot develop into the next life-cycle stage or infect the vector for vector-transmitted parasites [7,8]. However, high parasite intensity may lead to host mortality before the parasite is transmitted [9]. Host–parasite compatibility would set the upper and lower thresholds of the parasite intensity in the host; within this range, the parasite can survive, reproduce, and transmit before host mortality. Hence, to understand the occurrence and transmission of infectious disease, it is essential to evaluate the compatibility between parasites and their hosts, determined by host specificity of the parasite [10] and susceptibility of the hosts [11].

Host specificity is to what extent a parasite colonizes the hosts it can infect at a given life stage [9]. Parasites vary greatly in host specificity; some have the ability to infect a large variety of host species in different geographical locations, while others are restricted to one or a small set of hosts [12,13,14]. For parasites that can infect multiple host species, the severity of infections can differ significantly among those host species due to variations in body condition and immunocompetence [15].

The formation and persistence of host–parasite associations rely on a sufficient level of compatibility, assessed by the prevalence in the host population and mean infection intensities in host individuals, reflecting the ability of the parasite to infect and reproduce within the host [16]. Therefore, accurate assessment of host–parasite associations, including the diversity, prevalence, and infection intensity of parasites, is crucial for studying disease ecology.

Here, I use avian haemosporidian parasites as a model system, review the history of assessing their association with avian hosts, address the challenges, and highlight the important milestones that provide new insights for understanding the evolution of host–parasite associations.

## 2. Avian Haemosporidian Parasites as a Classical Model System

Avian malaria (*Plasmodium*) and related haemosporidian parasites (*Haemoproteus* and *Leucocytozoon*) are transmitted by dipteran vectors to thousands of bird species worldwide, causing infectious disease, reduced fitness [17], decreased life span [18], and even mortality [19]. As an ancestral close relative to human malaria but with much higher diversity, avian haemosporidian parasites have long been a classical system for investigating the biology and transmission of protozoan parasites [20]. Although rodent malaria was a popular model in earlier decades, avian haemosporidian parasites re-emerged as a model system in recent years for ecological and evolutionary studies on wildlife diseases due to their advantages of high diversity, universal distribution, and ease of access both in the field and laboratory [21]. Up till now most of the studies have focused on bird hosts due to the difficulty in matching parasites to the insect vectors [22]. Haemosporidian parasites undergo the agamic stages of their life cycle in birds, including exoerythrocytic merogony and the development of sexual stages in the blood cells, producing gametocytes that are infective for the vectors [23]. Current identification methods are mainly based on analyses of blood-stage parasites, i.e., merozoites and gametocytes for *Plasmodium* parasites, but only gametocytes for *Haemoproteus* and *Leucocytozoon* due to the lack of merogony in in erythrocytes. Blood samples collected from infected birds may also contain exoerythrocytic meronts before invading red blood cells or sporozoites just imported by vectors; both can be detected by sensitive molecular methods, but absent in blood smears.

For avian haemosporidian parasites, prevalence can be estimated by the proportion of infected individuals in the host population, while infection intensity is defined as the percentage of infected red blood cells in the bird blood sample [24]. Birds normally experience an acute stage with high infection intensity shortly after being infected and then maintain chronic infection for many years or even lifelong, with fluctuant but relatively lower infection intensity [23].

**Figure 1:**
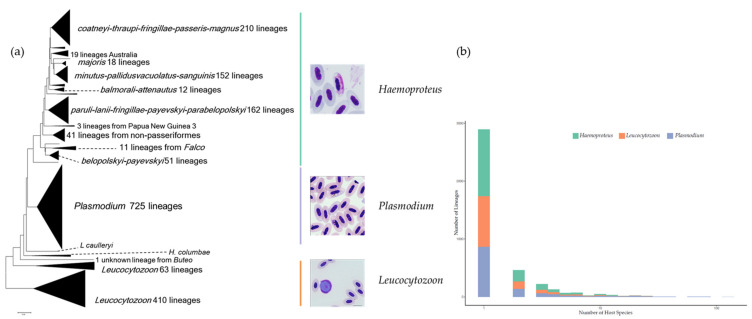
Phylogenetic pattern (**a**) and host diversity (**b**) of avian haemosporidian lineages. Data extracted from MalAvi database Version 2.4.9 (http://130.235.244.92/Malavi/index.html; Accessed on 28 April 2021). Microscopic images presenting the detectable life stages of avian haemosporidian parasites—gametocytes for all three genera and merozoites for *Plasmodium*.

From the very first discovery of avian haemosporidian parasites in blood smears, microscopy was the leading method for identification and quantification until the era of molecular approaches. Parasites belonging to different genera can be distinguished by their common features: presence of hemozoin pigment with erythrocytic merogony corresponds to *Plasmodium*; pigment present with no erythrocytic merogony implies *Haemoproteus*, while parasites with no pigment present and no erythrocytic merogony should belong to *Leucocytozoon* (Figure 1a). Based on a set of morphological characteristics of gametocytes and meronts in host erythrocytes, more than 250 morphospecies have been described [23]. By counting the number of parasites in hundreds of fields for each blood smear, microscopy can be very accurate and cost-effective in assessing infection intensity. In cases of mixed infection (i.e., two or more different parasite species infecting the same host individual simultaneously), which is common in wild birds, microscopy can identify each parasite species and assess their intensities separately [25]. Morphological identification based on microscopy has paved the way for theoretical and experimental studies on host–parasite associations and is still considered to be the gold standard for quantification of infection intensity today.

However, efficient microscopy requires experienced operators and high-quality blood smears with sufficient numbers of parasites covering most (if not all) developmental stages in red blood cells [26], making it a demanding task for beginners in many research groups. Moreover, when studying natural infections, wild-caught birds are mostly in the phase of chronic infection, as those suffering from acute infections are not active [17] or are already dead from the disease [27]. The low intensity of parasites in chronic stages often falls below the detection limit of microscopy (approximately 1 parasite per 10,000 erythrocytes) and results in underestimation of prevalence and infection intensity [28]. In addition, cryptic species with similar or no notable morphological characteristics cannot be distinguished by scanning blood smears [29], especially *Plasmodium* parasites with high cryptic diversity [30]. All these limitations have hampered further investigation of the global pattern of avian haemosporidian parasites.

## 3. Advances in Molecular Era

While it previously has been difficult to study the associations between avian haemosporidian parasites and their hosts due to many obstacles [31], the growing modern molecular technologies have provided various opportunities for the identification and quantification of this important group of parasites and offered the potential to answer longstanding questions on the ecology and evolution of host–parasite associations, including the adaptation of parasites to multiple hosts [16], the impact of chronic infections [18], and the role of biotic and abiotic environmental factors in shaping host–parasite associations [32,33].

### 3.1. Molecular Approaches for Taxonomic Identification

Since the first molecular identification method for avian haemosporidian parasites was published [34], a number of studies have been carried out, and various protocols have been developed in parallel [35,36,37,38]. The application of molecular methods opened a new door for detecting and characterizing avian haemosporidian parasites and soon replaced the dominant position of microscopy for its high sensitivity, time savings, and enabling more precise taxonomic classification [39], indicating the beginning of the molecular era.

A segment of the cytochrome b gene located in the mitochondrial genome of the parasites was chosen as the barcoding sequence, which can be amplified by several PCR assays, with products ranging between 479 and 533 bp. Parasites with at least one base pair difference in the barcoding sequence were defined as unique lineages [40]. The majority of early studies focused on describing the lineage diversity of haemosporidian parasites in one or a few particular bird communities [41], as a result, the recognized diversity has increased 10-fold over the past 20 years. To date, more than 4000 lineages have been defined, the phylogeny of these lineages based on barcoding sequence clearly clustered in three clades, each corresponding to a genus, indicating the consistence in taxonomic identifications based on morphological and molecular methods (Figure 1a). Lineages with similar morphological characters are defined as the same morphological species, but deeper investigation demonstrated that in some cases they correspond to cryptic species [29,42], addressing the importance of molecular evidence in investigating host–parasite associations. Molecular data has also put forth several phylogenetic hypotheses among avian haemosporidian parasites and other blood parasites; although frequently inconsistent, all of them have supported the traditional assumption that human malaria parasites are of avian origin [20]. 

Molecular-based studies are illustrating an ever-growing picture of the host range and diversity hotspots of avian haemosporidian parasites on a global scale [43]. The vast majority of recorded lineages appear to be specialists with only one or a few recorded host species, while some generalist lineages (mostly *Plasmodium*) have been recorded in more than 50 different host species (Figure 1b). With an increasing number of reports on heterogeneous patterns of host–parasite associations, the research priorities subsequently turned to exploring the formation and evolution of those associations [44]. Ellis et al. [45] identified a phylogenetic pattern, indicating that lineages in the same clade of phylogenetic tree represent similar levels of host specificity (in terms of the number of host species a parasite can infect), while Fecchio et al. [33] suggested that environmental factors such as climate are important drivers in the evolution of host–parasite associations. Studies at the community level have revealed the roles of various evolutionary events that may shape host–parasite associations, including cospeciation and host shift [46,47,48]. However, how multiple hosts contribute to the evolution of generalist parasites remains unknown.

Given that conventional PCR-based methods can determine prevalence and diversity of avian haemosporidian parasites simultaneously, it was employed as the solitary method in many studies, and prevalence was used as the only index to estimate the compatibility between avian haemosporidian parasites and their hosts in the majority of studies. However, this may lead to incorrect inferences of host–parasite associations due to three causes.

First, mixed infections, which are common in the wild, are often underestimated by most of the described molecular assays [49], probably owing to the unpredictable selective amplification and the uneven ratio among different parasite lineages [50]. The recently developed multiplex PCR assay managed to simultaneously identify avian haemosporidian parasites in different genera [51], representing a significant improvement in the molecular detection of mixed infections, yet lineages within the same genus remain undistinguishable.

Second, molecular identifications cannot distinguish abortive infections from real host–parasite associations. When a parasite occasionally encounters host species to which it is not optimally adapted, it may survive for a short period but cannot complete its life cycle [52]. Such abortive infections can be identified by the absence of gametocytes (the life stage in which the parasite can be transmitted to insect vectors) or extremely low infection intensity. Unfortunately, neither life-stage information nor infection intensity data can be acquired in conventional PCR analysis. Considering that all positive samples were equally treated, the observed host range of parasites may be overestimated.

Third, the lack of infection intensity data restricted our understanding of the compatibility between parasites and their hosts. For generalist parasites with the ability to infect multiple host species, it is unlikely that all hosts harbour equal amounts of parasites [53,54]. Instead, they are often better adapted to a handful of main host species than others due to variation in host immune systems and host–parasite coevolution, resulting in dramatic differences in levels of infection intensities [16,55]. Therefore, without information on the relative contributions of parasites among different host species provided by infection intensity data, one can barely reveal the evolutionary history of generalist parasites.

Once the importance of infection intensity was recognized, the combination of microscopy and molecular methods was frequently employed, but owing to variations in the sensitivity of assays [56] and examiner experience [57,58], these studies often ended up with unconvincing results in evolutionary studies on host–parasite associations and ecological studies comparing differences among habitat niches [59]. Ecological and evolutionary studies on host–parasite associations call for more accurate and sensitive quantification technology.

### 3.2. The Milestone in Molecular Quantification

Advances in molecular techniques and the availability of real-time quantitative PCR (qPCR) have provided a novel approach for parasite diagnosis in various materials, including bird blood, tissue samples, and vector samples. Compared with conventional PCR, the qPCR method is faster, more sensitive, and requires less material in each reaction [60]. Most importantly, qPCR enabled rapid quantification of avian haemosporidian parasites in DNA samples by monitoring the fluorescence signal during the PCR, which is proportional to the quantity of target genes.

**Figure 2:**
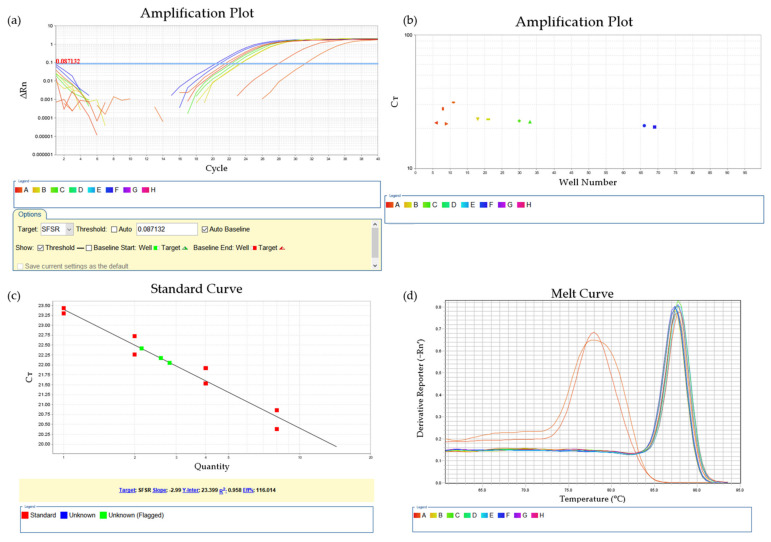
An example of the reported result of a real-time qPCR reaction performed on a 7500 Real-Time PCR instrument (Applied Biosystems, California, USA). This reaction plate includes two unknown samples, four standard samples, and two non-template-controls (NTCs). (**a**) Amplification curve with threshold line; (**b**) Automatically calculated Ct value for each sample; (**c**) Standard curve with amplification efficiency; (**d**) Melt curve, non-specific amplifications can be recognized based on the position of peaks.

During the qPCR reaction, the fluorescence signal of dyes (bound with all double-strain DNA) or probes (bound with the target sequence) was captured after each amplification cycle, and in the end, an amplification curve was generated for each sample together with a threshold line (Figure 2a). The number of cycles during which the signal reached the threshold was recorded as the Ct value of the sample (Figure 2b). By comparing Ct values, the relative amounts of parasite in the initial templates can be acquired. For more accurate quantification, serially diluted standard samples with known infection intensities can be included to generate a standard curve and amplification efficiency of the reaction (Figure 2c).

Another advantage of qPCR is the reduction of false positives by melting curve analysis (Figure 2d). Immediately after the PCR, the temperature was gradually increased, and the fluorescence signal was monitored during the whole process. As the melting temperature (the temperature at which DNA strands separate) varies with the length and base sequence of the DNA, primer–dimers and nonamplification products can easily be distinguished from the melting curve [61].

For sufficient amplification efficiency and specificity, the ideal amplicon of qPCR should be a GC-rich fragment ranging between 70 and 200 bp. Given that the genome size of the bird is more than 50 times larger than the genome size of the parasite [62], it may incidentally contain a segment that is similar to the targeting sequence; additionally, GC-rich fragments are scarce in the parasite genome [63]. Moreover, due to the tremendous difficulties in genomic sequencing of avian haemosporidians, the available data were restricted to mitochondrial genes and sporadic nuclear gene sequences from a few lineages until recently [64], making molecular quantification of avian haemosporidian parasites even more difficult. In spite of the difficulties, several qPCR protocols were established, mostly targeting a fragment within the barcoding sequence [65] or the more conserved ribosomal-RNA region [56,66,67], and previously developed primers were employed in qPCR. To minimize the bias caused by the concentration of DNA templates, a single copy nuclear sequence from the bird genome was used as a housekeeping gene in follow-up studies, enabling more accurate estimation of infection intensities [59]. Nevertheless, mixed infections, which are a common barrier for molecular identification methods, remain unresolved in general qPCR. In cases of mixed infections, only total infection intensity can be obtained.

After continuous trying, the first genome-wide sequences of avian haemosporidian parasites were published [68], followed by more transcriptome [69,70] and genome sequences [71,72], paving the way for assessing specific host–parasite associations in multiple dimensions and triggering a second wave of qPCR development, targeting one or a set of focal haemosporidian parasites [16,51] to settle more specific ecological and evolutionary issues.

Molecular quantification analysis supported the previous assumption that incongruences between microscopy and conventional PCR results occur mainly in samples with low infection intensity and shows its own advantages. Compared with the traditional microscopy method, qPCR is much more time-saving and more sensitive [58,73]. Compared with conventional PCR, infection intensity data provide evidence for exploring the compatibility between avian haemosporidian and their hosts [16].

Comparisons of infection intensity in different host species suggested that generalist parasites are better adapted to a few main hosts, which they may have encountered more frequently during evolution [16]. By monitoring the dynamics of infection intensities, a clear pattern of spring relapse was detected in many bird species [74], and late May to June was recognized to be the peak time of infection, during which most bird species breed [75,76] and the density of active insect vectors increases coincidentally [77]. Genomic data have enabled specific qPCR targeting focal haemosporidian lineages, which were used to test the correlation between mixed-infected parasites [78]. With all these findings, the achievement of real-time quantitative PCR is another significant milestone that not only enables more rapid and sensitive diagnosis of avian haemosporidians [58] but also sheds light on how host–parasite associations were formed and evolved [16].

### 3.3. From Relative to Absolute Quantification

Although qPCR has enhanced the quantification of infection intensities to a great extent, the results largely rely on laboratory-based standard samples, which may degrade over time. Due to the constraint of standard samples, a comparison of infection intensity assessed in different laboratories or across many years is still inaccessible [79].

**Figure 3:**
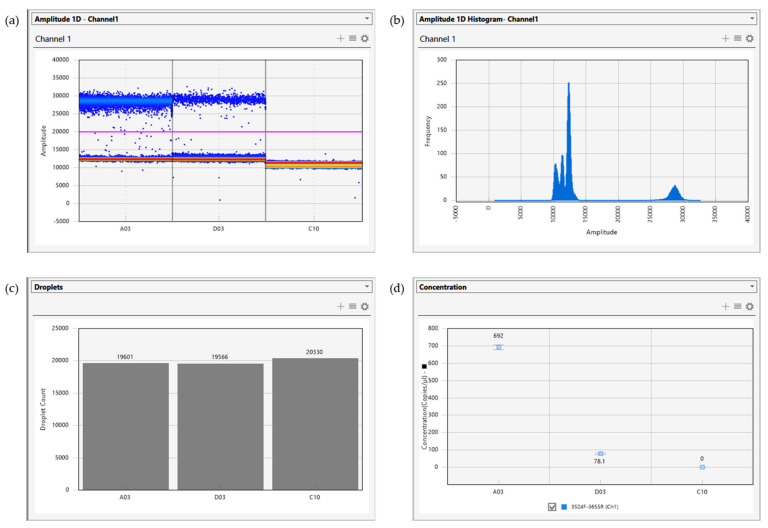
An example of ddPCR output from QX200TM Droplet Reader (Bio-Rad California, CA, USA), presenting samples with high haemosporidian quantity (A03), low quantity (D03), and the NTC (C10). (**a**) Distribution pattern of droplets; the ‘rain’ close to the threshold line represents false positives caused by primer dimer or nonspecific amplifications; (**b**) Histogram of droplets. (**c**) Total count of droplets in each PCR well; (**d**) Concentration of target gene fragments in each assessed sample, calculated with the default setting of the droplet reader. Adapted and reprinted with permission from ref. [79]. Copyright © 2020, Xi Huang et al.

The recently developed digital droplet PCR (ddPCR) technique offers a potential solution to this problem. In a ddPCR reaction, the whole system, including standard PCR reagents and fluorescence dye, was divided into 20,000 random droplets using water–oil emulsion technology, and amplifications were accomplished in each of the nanolitre-sized droplets independently. Positive or negative droplets were determined by the presence or absence of a fluorescence signal, and the absolute copy number of the target gene in initial samples was calculated using Poisson analysis (Figure 3).

Unlike qPCR, the fluorescent signal in the ddPCR reaction system was not related to the amount of amplified target gene fragments. A droplet can be defined as positive as long as amplification occurred, even if the efficiency was low. Hence, ddPCR can be more sensitive and less reliant on the quality of initial samples, making it an ideal method when the target gene is only a tiny fraction of the sample. Besides, independent amplifications in droplets are equivalent to millions of times of repeated experiments, minimizing the randomness of PCR caused by various factors, such as background DNA; PCR inhibitors including haemoglobin and alcohol; consumption of enzymes, etc. These advantages have been confirmed by previous studies on disease diagnosis [80] and therapeutic [81] quantification of protozoan parasites from faecal samples [82] and human malaria from blood samples [83].

Although just introduced to avian haemosporidian parasite studies, ddPCR has already exhibit strong advantages in absolute quantification of infection intensities independent of standard samples. With the identical reaction system (i.e., same samples and same primers), ddPCR represented higher sensitivity and repeatability than other molecular methods, especially when infection intensity was low [79].

The big step of absolute quantification facilitated investigating the evolution of host–parasite associations, by enabling comparisons in infection intensities on larger scales, namely, monitoring annual variations on time scale and investigating infection patterns of widely distributed parasites on a space scale.

As an emerging technique, ddPCR inevitably has its weakness. Non-specific amplifications and primer dimers could lead to false positive droplets and form a ‘rain’ function in the droplet histogram. As the amplicon of ddPCR is short (up to 120 bp), the ‘rain’ may occasionally be difficult to distinguish and cause an overestimation in infection intensity. Future studies with optimized protocol or more specific primers may help with resolving this problem.

### 3.4. The Emergence of New Technologies for Future Exploration

The emergence of new technologies has facilitated more rapid and sensitive diagnostics in various taxa, including human malaria and other mammalian parasites, while studies on avian haemosporidians are still lagging behind [64]. Being restricted by the biology of the host (i.e., nucleoid red blood cells of avian hosts) and life history traits (i.e., no erythrocytic merogony for *Haemoproteus* and *Leucocytozoon*; no pigment present for *Leucocytozoon*), some effective methods such as fluorescence in situ hybridization are almost impossible to accomplish when studying avian haemosporidian parasites, while others may provide potential opportunities for future exploration (Table 1).

Multiple techniques have been developed and widely used in human malaria diagnosis during the last couple of years, including nuclear magnetic resonance (NMR) and various magnetic field sensors [84], which enables the identification of extremely low parasite load based on certain atomic nuclear signals [85], and the rotating-crystal magneto-optical detection (RMOD) method, which makes use of the increased magnetic susceptibility of infected red blood cells [86]. If these methods can be applied to avian haemosporidian parasites, they can be useful tools for rapid diagnosis in field studies. Haemoglobin electrochemical detection [87,88] could provide more opportunity to explore the morphological characters, which are currently lacking in avian haemosporidian parasite studies. Future exploration on mature methods in close related parasites would indeed inspire advances in understanding the associations between avian haemosporidian parasites and their hosts.

## 4. Conclusions

As one of the oldest model systems for investigating disease evolution, avian haemosporidian parasites have been studied for almost a century, during which technical advances have brought about various methods for respective scopes (Table 2) and together broadened our understanding of host–parasite associations to settle modern issues.

Briefly, all these methods are able to identify haemosporidian, i.e., to determine the presence or absence of infection. Among them, microscopy remains the gold standard for morphological identification and absolute quantification of infection intensity, despite its time cost and relatively lower sensitivity. For researches on avian haemosporidian parasites, microscopy remains the only method that can provide morphological characteristics in different life stages, which is crucial for species definition. Conventional PCR is the primary choice for taxonomic identification and phylogenetic analysis, as both qPCR and ddPCR have forsaken sequencing processes. However, due to the lack of infection intensity data, conventional PCR is not favoured in studies focusing on certain parasite populations or communities. None of the molecular methods can confidently distinguish abortive infections, but samples with low infection intensity detected by qPCR and ddPCR can be suspected as abortive infections. Both molecular quantification methods can be used to investigate infection patterns but are applicable to different scales. When studying in-population variations in infection intensity, qPCR works well with relative quantification and standard curves, but for comparisons across larger scales or more accurate quantities, we call for the more conserved ddPCR method.

To date, there are still ongoing challenges in the quantification of avian haemosporidian parasites. First, the copy number of mitochondrial genomes in a haemosporidian gametocyte remains unclear therefore, the copy number of mitochondrial genes assessed by qPCR or ddPCR could not simply convert to parasite quantity unless verified by microscopy results. In other words, rapid absolute quantification of avian haemosporidians has not been achieved yet. However, the progressing deep learning techniques might contribute to future work by improving the efficiency of blood smear scanning. Although taxonomic identification might still be difficult, the application of machine learning can help with real-time detection. Second, for *Plasmodium* parasites (but not *Haemoproteus* or *Leucocytozoon*) with erythrocytic merogony in their life cycles, some erythrocytes may harbour plenty of merozoites or meronts, leading to a grossly overestimated infection intensity. Meanwhile, such differences in life cycles hampered comparisons of infection intensity across parasite genera. Future genomic advances may provide candidate markers with a known copy number for more accurate quantification.

From the past until now, studies on avian haemosporidian parasites have overcome many challenges, thanks to the boost of technology, which is catching up much more rapidly than expected. The gradually expanding database and increasing resources of genomic data can serve as backbones for designing more credible diagnosis protocols and further revealing the ecology and evolution of host–parasite associations. Ecological and evolutionary studies on host–parasite associations are accelerating in a promising way. We are still moving on.

## Figures and Tables

**Table 1:** Emerging technologies in other parasites and their potential for studying avian haemosporidian parasites.

Method	Summary	Potentials	Restriction	Ref.
photoacoustic PA-SAW	Diagnosis of malaria at early stage based on photoacoustics signal.	Applicable in *Plasmodium*	Applicable at ring-stage which inly present in *Plasmodium*	[89]
Magneto-optical diagnosis	Detect hemozoin in very small concentrations based on crystal structure.	Worth trying	Nucleoid red blood cell may be inhibitor.	[86]
Magnetic Field Sensors	Detect infection based on the increased magnetic susceptibility of infected red blood cells.	Potentially useful		[84]
Haemoglobin electrochemical	Detect infection based on transfer characteristics of haemoglobin.	Potentially useful	Unfit for *Leucocytozoon* due to lack of pigment	[87,88]

**Table 2:** Comparison of the most widely used methods in studying avian haemosporidian parasites.

Method	Scope	Fields of Application	Advantages	Limitations	Ref.
Microscopy	Diversity Infection intensity	Morphological identification Life stage description	Hardly any false positive; Identify mixed infections	Time-consuming Low sensitivity	[23]
Conventional PCR	Diversity	Molecular identification Phylogenetic analysis	Taxonomic classification; Cost-effective	Underestimate mixed and abortive infections	[34,36,37,38]
qPCR	Infection intensity	Relative quantification Infection pattern in small scale	Rapid diagnosis; Eliminate false-positive	Taxonomy undefinable; Rely on standard	[16,59,66,67]
ddPCR	Infection intensity	Absolute quantification Infection pattern in large scale	Sensitive and repeatable; Low demand in samples	Taxonomy undefinable; Costly	[79]

## Data Availability

Not applicable.
